# Supplementary material for: A conserved C. elegans zinc finger homeodomain protein, ZFH-2, is continuously required for the structural integrity and function of the alimentary tract and gonad
Source: Development. 2026 Apr 13;153(7):dev205502. doi: 10.1242/dev.205502 (PMC13078641; doi:10.1242/dev.205502)
Supplement: Supplementary information [file develop-153-205502-s1.pdf]

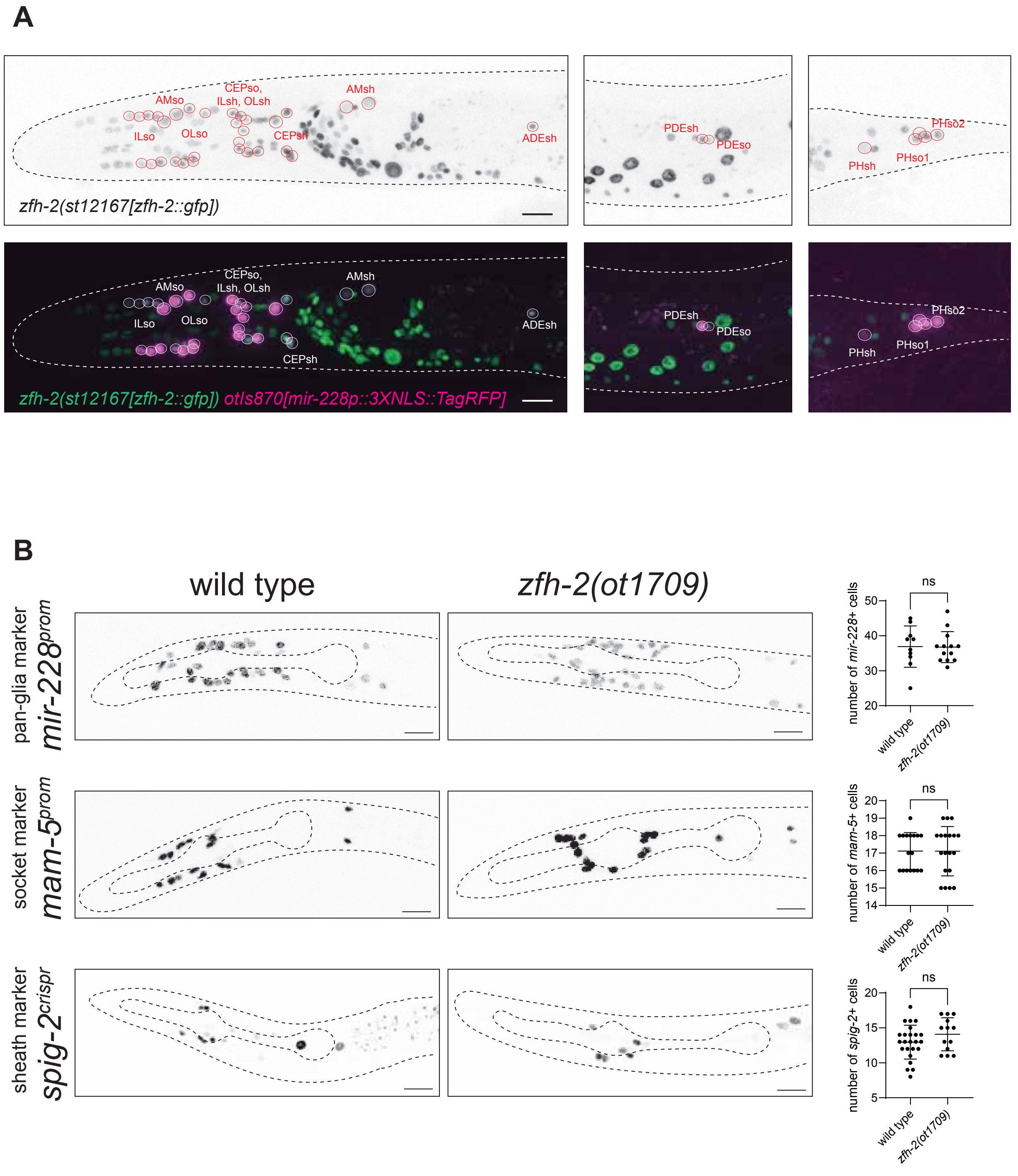

Fig. S1. **ZFH-2 is expressed in all ectodermal glia of *C. elegans* but is dispensable for specification of glial fates. (A)** A *gfp*-tagged allele of *zfh-2 (st12167[zfh-2::GFP + loxP + unc-119(+) + loxP])* reveals expression in all ectodermal glial cells, identified by the pan-glial marker *otIs870[mir-228p::3xNLS::TagRFP-T]*. **(B)** The *zfh-2* null allele *ot1709* does not significantly decrease the number of cells expressing the pan-glia marker *otIs870[mir-228p::3XNLS::TagRFP-T]* (Miska et al., 2007; Wang et al., 2024), the socket marker *otIs857[mam-5p2::3xNLS::TagRFP-T],* and the pan-sheath marker *spig-2(syb6670[spig-2::sl2::gfp::h2b])*(Aguilar and Hobert, 2024) in arrested L1 larvae. Statistical analysis was performed using unpaired t-test. ns: not significant.

Development • Supplementary information

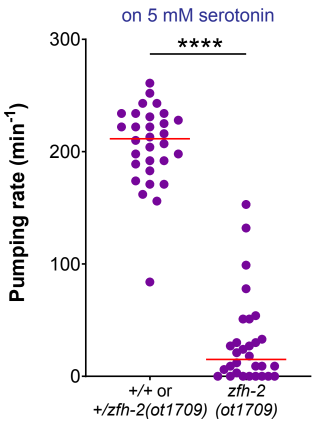

Fig. S2. Serotonin does not rescue the pumping defects of *zfh-2* mutant. Pharyngeal pumping rate of *zfh-2(ot1709)* L1 larval stage animals in the presence of 5 mM serotonin. Horizontal line in the middle of data points represents median value of biological replicates. **** $P < 0.0001$ in Mann-Whitney test.

Development • Supplementary information

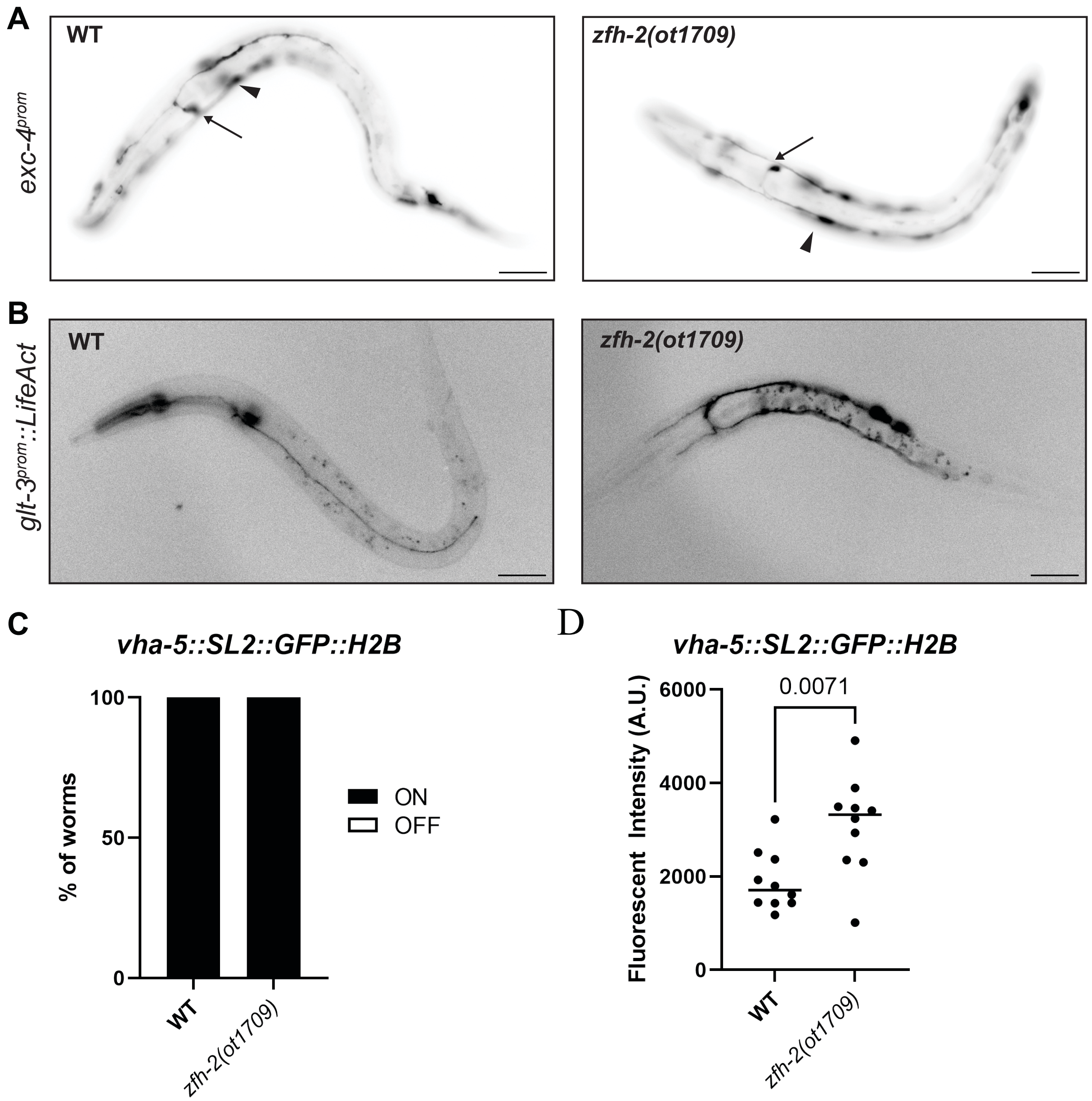

Fig. S3. *zfh-2* null mutants show no obvious defects in the excretory cell (A) An extrachromosomal array carrying an *exc-4prom::GFP* promoter fusion construct (*otEx669[exc-4p::GFP + rol-6(su1006)]*) in wild type and *zfh- 2* null animals shows normal excretory canal morphology. Arrow is canal, arrowhead is hypodermal cell expression. **(B)** When expressed under a *glt-3* excretory cell specific promoter fragment (*arIs195[glt-3p::LifeAct::TagRFP]*), the actin reporter *LifeAct::TagRFP* shows similar excretory canal morphology in wild type and *zfh-2* null animals. **(C)** Animals homozygous for *zfh-2* null alleles retain expression of the excretory cell marker *vha-5(syb6835[vha-5::sl2::gfp::h2b])*. **(D)** Expression strength of the excretory cell marker *vha-5(syb6835[vha-5::sl2::gfp::h2b])* is not decreased in animals homozygous for *zfh-2* alleles.

Development • Supplementary information

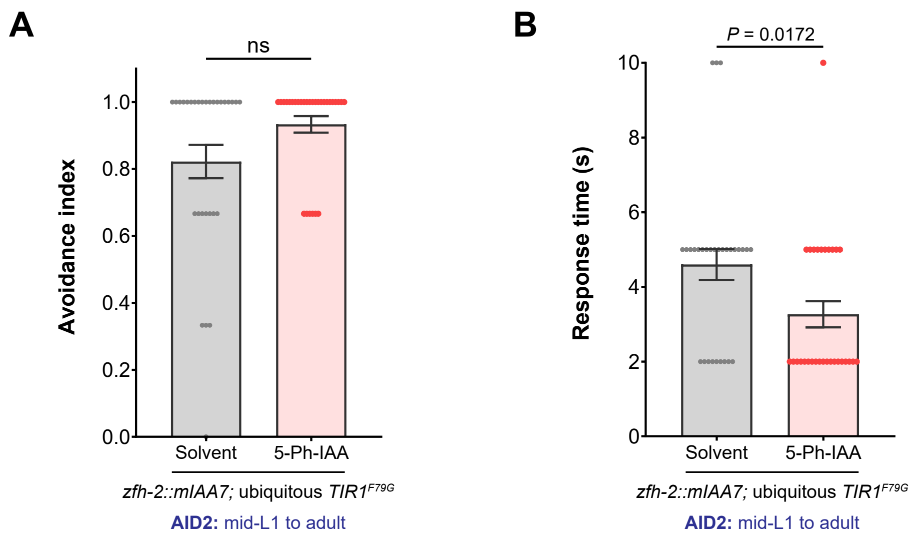

Fig. S4. Postembryonic removal of ZFH-2 protein does not affect octanol avoidance behavior Avoidance index **(A)** and response time **(B)** of *zfh-2(syb10278); osIs158[eft-3p::TIR1(F79G)]* adults to 100% octanol. Animals were treated with either solvent (ethanol) or 100 µM 5-Ph-IAA starting at mid-L1 stage. Bars represent mean ± SEM. Data points represent individual animals. Denoted *P* value and not significant (ns) are from Mann-Whitney tests.

Development • Supplementary information

Table S1. Expression pattern of ZFH-2::GFP protein in late larval/young adult stage animals. Bold and underlined indicates that no homeobox identity regulator has yet been described for this neuron class. * indicates that expression in this neuron was previously overlooked.

Neurons	Anterior ganglion	IL1, OLQ, URY
	Other head ganglia	ADE, **ADF**, AFD, AIB, AIM, AIY, AIZ, ALA, ASG, ASH, **ASJ**, AVA, AVE, AVJ, AVL, AWB, CEP, FLP, RID, RIG, **RIM**, RIR, RIV, RMD, RMH, SAA, SAB, SIA, SIB, SMB, SMD
	Midbody	BDU, PDE, PVD, PVM, CAN*
	VNC	AS, DA, DB, VA, VB
	Tail	**DVA**, DVC, LUA, PDA, PDB, PHA, PHB, PQR, PVN
	Pharynx	NSM
Glia	All sheath glia	
	All socket glia	
	Not in GLR glia	
Excretory system	Canal cell	
	Gland cells	
	Duct cell	
	Pore cell	
Gonad	Spermatheca	
	Spermatheca-uterine valve	
	Gonadal sheath cells	
Vulva	Subset of vulval cells (vulE, vulF)	
Alimentary tract	Subset of pharyngeal muscle cells (pm1, pm2, pm7V, pm8)	
	Subset of pharyngeal epithelial cells (e1)	
	Subset of pharyngeal neurons (NSM only)	
	Pharyngeal-intestinal valve (VPI)	
	Not in hmc	
	Intestine: posterior intestinal cells only	
	Rectal valve cells (virL, virR)	
	Rectal gland cells (rect_D, rect_VL, rect_VR)	
	Rectal epithelial cells (U, Y, B, F, K, K')	
Body wall muscle	Not expressed	
Hypodermis	Not expressed	

Table S2: Strain list.

**Strain**	***Mutant or knock-in***	**Array**	**DNA on array**	**Reference**
OH20426	*zfh-2(ot1812[zfh-2::gfp::loxP][*st12167]) I*			This study
OH20145	*zfh-2(ot1709)/tmC20[unc-14(tmIs1219) dpy-5(tm9715)] I*			This study
EG4887	*unc-119(ed3) III*	*oxIs322*	*myo-2p::mCherry::H2B, myo-3p::mCherry::H2B, Cbr-unc-119(+)*	PMID: 18953339
OH15814	*him-5(e1490) V; dmd-4(ot935[dmd-4::gfp]) X*			PMID: 33021200
PHX4257	*eat-4(syb4257[eat-4::T2A::GFP::H2B]) III*			PMID: 34415309
PHX4491	*unc-17(syb4491[unc-17::T2A::GFP:H2B]) IV*			PMID: 35324425
PHX5463	*ins-6(syb5463[ins-6::SL2::gfp::H2B]) II*			PMID: 36178933
PHX6486	*cat-1(syb6486[cat-1::SL2::gfp::H2B]) X*			PMID: 39422452
PHX8255	*cat-2(syb8255[cat-2::sl2::gfp::h2b]) II*			PMID: 39422452
PHX6451	*tph-1(syb6451[tph-1::SL2::gfp::H2B]) II.*			PMID: 39422452
PHX7768	*tdc-1(syb7768[tdc-1::sl2::gfp::h2b]) II*			PMID: 39422452
OH15623		*otIs706*	*nlp-12p::tagRFPintrons*	This study
PHX10278	*zfh-2(syb10278[zfh-2::mIAA7::wrmScarlet-I3::mIAA7]) I*			This study
HS3528		*osIs158 II*	*eft-3p::TIR1(F79G)::mRuby*	PMID: 34865044
OH19717		*otIs935 V*	*UPNp:TIR1(F79G)::mTurquoise2:tbb-2 3' UTR, unc-122p:mCherry:unc-54 3' UTR*	This study
OH20419	*zfh-2(syb10278[zfh-2::mIAA7::wrmScarlet-I3::mIAA7]) I*	*otSi4 II*	*myo-2p::TIR1(F79G)::mRuby::unc-54 3'UTR *ieSi60*	This study
FX17732	*zfh-2(tm310)/hT2[bli-4(e937) let-?(q782) qIs48]*			National BioResource Project
FX22720	*zfh-2(tm12720) I*			National BioResource Project
OH20425	*zfh-2(syb10278 ot1787[zfh-2$^{\Delta HD2}$])/tmC20[unc-14(tmIs1219) dpy-5(tm9715)] I*			This study
OH20490	*zfh-2(syb10278 ot1797[zfh-2$^{\Delta HD3}$]) I*			This study
OH20491	*zfh-2(syb10278 ot1787[zfh-2$^{\Delta HD2}$] ot1798[zfh-2$^{\Delta HD3}$])/tmC20[unc-14(tmIs1219) dpy-5(tm9715)] I*			This study
OH20517	*zfh-2(syb10278 ot1807[zfh-2a M133>STOP] ot1808[zfh-2a M233>STOP M234>STOP) I*			This study

Development • Supplementary information

OH20518	*zfh-2(syb10278 ot1809[zfh-2d M50>STOP]) / tmC20[unc-14(tmIs1219) dpy-5(tm9715)] I*			This study
OH17760		*otIs870*	*mir-228p::3XNLS::TagRFP*	PMID: 18085825, 39422452
OH17400		*otIs857*	*mam-5prom2::3xNLS-TagRFP-T*	PMID: 32696701, 40950173
PHX6670	*spig-2(syb6670[spig-2::sl2::gfp::h2b]) V*			PMID: 38304163
CM1360		*guIs30*	*clh-4::gfp*	PMID: 19921263
QN305		*arIs195*	*glt-3p::LifeAct::TagRFP*	PMID: 27697907
OH1277		*otEx669*	*exc-4::gfp; rol-6(su1006)*	PMID: 14684823
AG408	*pezo-1(av146[gfp::pezo-1]) IV*			PMID: 32490809
